# Disparate Impact of Butyroyloxymethyl Diethylphosphate (AN-7), a Histone Deacetylase Inhibitor, and Doxorubicin in Mice Bearing a Mammary Tumor

**DOI:** 10.1371/journal.pone.0031393

**Published:** 2012-02-23

**Authors:** Nataly Tarasenko, Suzanne M. Cutts, Don R. Phillips, Aida Inbal, Abraham Nudelman, Gania Kessler-Icekson, Ada Rephaeli

**Affiliations:** 1 Felsenstein Medical Research Center, Sackler Faculty of Medicine, Tel-Aviv University, Beilinson Campus, Petach-Tikva, Israel; 2 Thrombosis and Hemostasis Unit, Rabin Medical Center, Beilinson Hospital, Petach-Tikva, Israel; 3 Department of Biochemistry, La Trobe University, Victoria, Australia; 4 Department of Chemistry, Bar-Ilan University, Ramat-Gan, Israel; Sanford Burnham Medical Research Institute, United States of America

## Abstract

The histone deacetylase inhibitor (HDACI) butyroyloxymethyl diethylphosphate (AN-7) synergizes the cytotoxic effect of doxorubicin (Dox) and anti-HER2 on mammary carcinoma cells while protecting normal cells against their insults. This study investigated the concomitant changes occurring in heart tissue and tumors of mice bearing a subcutaneous 4T1 mammary tumor following treatment with AN-7, Dox, or their combination. Dox or AN-7 alone led to inhibition of both tumor growth and lung metastases, whereas their combination significantly increased their anticancer efficacy and attenuated Dox- toxicity. Molecular analysis revealed that treatment with Dox, AN-7, and to a greater degree, AN-7 together with Dox increased tumor levels of γH2AX, the marker for DNA double-strand breaks and decreased the expression of Rad51, a protein needed for DNA repair. These events culminated in increased apoptosis, manifested by the appearance of cytochrome-c in the cytosol. In the myocardium, Dox-induced cardiomyopathy was associated with an increase in γH2AX expression and a reduction in Rad51 and MRE11 expression and increased apoptosis. The addition of AN-7 to the Dox treatment protected the heart from Dox insults as was manifested by a decrease in γH2AX levels, an increase in Rad51 and MRE11 expression, and a diminution of cytochrome-c release. Tumor fibrosis was high in untreated mice but diminished in Dox- and AN-7-treated mice and was almost abrogated in AN-7+Dox-treated mice. By contrast, in the myocardium, Dox alone induced a dramatic increase in fibrosis, and AN7+Dox attenuated it. The high expression levels of c-Kit, Ki-67, c-Myc, lo-FGF, and VEGF in 4T1 tumors were significantly reduced by Dox or AN-7 and further attenuated by AN-7+Dox. In the myocardium, Dox suppressed these markers, whereas AN-7+Dox restored their expression. In conclusion, the combination of AN-7 and Dox results in two beneficial effects, improved anticancer efficacy and cardioprotection.

## Introduction

Targeting histone modifications for therapeutic purposes has been extensively investigated [1]. Studies have shown that the pharmacological inhibition of histone deacetylase (HDAC) activity may be beneficial for a variety of diseases [2], [3]. Our group has developed and studied several histone deacetylase inhibitory prodrugs of butyric acid, which release acids and aldehydes upon intracellular hydrolytic degradation [4]–[6]. These compounds were found to modulate gene expression and induce histone hyperacetylation and apoptosis of cancer cells in vitro and in vivo [7]–[9]. Butyroyloxymethyl-diethyl phosphate (AN-7), which releases butyric acid, formaldehyde and phosphoric acid, was the most potent of the prodrugs evaluated and displays significant in vivo activity. At doses of 25–50 mg/kg, AN-7 inhibits tumor growth, angiogenesis, and metastasis in several mouse cancer models [9], [10]. The main advantages of AN-7 are its specific activity against cancer cells and its synergistic interaction with commonly used anticancer agents, such as anthracyclines and antibodies against epidermal growth factor receptor (HER)-2 [11], [12].

Doxorubicin (Dox) is one of most effective drugs currently available for the treatment of neoplastic diseases. However, its use is complicated by dose-limiting cardiotoxicity and the development of multi-drug resistance [13]. One of the contributors to Dox-induced cardiotoxicity is the formation of reactive oxygen species (ROS), a critical mediator of myocardial damage, which, with time, can lead to cardiomyopathy leading to heart failure [14].

We have shown that AN-7 attenuates Dox-activated increase in reactive oxygen species (ROS)-dependent death of cardiomyoblasts and cardiotoxicity in mice. Conversely, in 4T1 mammary carcinoma cells, AN-7 interacts synergistically with Dox to induce ROS-dependent cell death [12], [15]. These opposing effects of AN-7 and Dox reflect the changes in the levels of phosphorylated histone γH2AX, a marker of DNA double-strand breaks (DSBs). There are also differences in the levels of c-Myc and angiogenic factors. In an in vivo study of AN-7 (50 mg/kg thrice a week for 3 weeks) and Dox (a single dose of 20 mg/kg) combination, the increased level of c-Myc in the heart was accompanied by an increased expression of the hypoxia-inducible factor (HIF)-1α [12]. HIF-1α controls the transcription of pro-angiogenic and cardioprotective factors, including vascular endothelial growth factor (VEGF), VEGF receptor, erythropoietin, and heme-oxygenase (HO-1) [16]. Accordingly, we observed that treatment of mice with AN-7 and Dox, elevated HO-1 expression in the myocardium and diminished blood levels of the pro-inflammatory cytokines, tumor necrosis factor (TNF)-α and interferon-γ [12].

AN-7 together with Dox treatment shows cell-type selective changes of key proteins that are involved in the control of viability, inflammation and angiogenesis. This demonstrates the specificity of this treatment. After showing the in vitro effects, the next important step was to demonstrate that these events occur simultaneously in tumor bearing animals. In this study, we explored the pro- and anti-survival changes, both the physiological and molecular changes which take place concomitantly in the tumors and the hearts of mice bearing 4T1 mammary tumors, following treatments with either Dox or AN-7 or AN-7+Dox.

## Materials and Methods

### Compounds and reagents

AN-7 was synthesized as described [17]. Doxorubicin hydrochloride, 2 mg/mL, was obtained from Ebewe Pharma Ges.m.b.H. (Unterach, Austria). The following antibodies were used: goat polyclonal anti-MRE11 (meiotic recombination 11 homolog A of *S. cerevisiae*, Santa Cruz Biotechnology, Santa Cruz, CA, USA); rabbit polyclonal antibodies for c-Myc (Cell Signaling, ck USA), low molecular weight fibroblast growth factor (lo-FGF Santa Cruz Biotechnology), HO-1 (Stressgen, Ann Arbor, MI, USA), cytochrome-c, HDAC5 (Cell Signaling, Danvers, MA, USA), HDAC1 (Sigma, St. Louis, MO, USA), c-Kit and Rad51 (Santa Cruz Biotechnology), Ki67 (Lab Vision, Fremont, CA, USA); mouse monoclonal antibodies for phosphor-H2AX, Ser 139, (γH2AX) (BioLegend, San Diego, CA, USA), vascular endothelial growth factor (VEGF, Santa Cruz Biotechnology), thrombospondin -1 (TSP-1) (Lab Vision, Fremont, CA, USA), HDAC2 (Abcam, Cambridge, UK), and actin (MP Biomedicals, Aurora, Ohio, USA). Secondary antibodies: horseradish peroxidase (HRP)-conjugated goat anti-rabbit IgG and HRP-goat anti-mouse IgG (Jackson ImmunoResearch Laboratories, West Grove, PA, USA); IRDye® 680 goat anti-mouse or anti-rabbit IgG (LI-COR Biosciences, Lincoln, NE, USA); biotinylated goat anti-rabbit and anti-mouse IgG-B (Santa Cruz Biotechnology).

### Cell cultures

The murine mammary carcinoma 4T1 (CRL-2539) and embryonic rat immortalized heart H9C2 (CRL-1446) cell lines were obtained from ATCC (Rockville, MD, USA). The U251 MG human glioma cell line was obtained from the University of California, San Francisco brain tissue bank, and normal human astrocytes (NHA) were obtained from Lonza International, Basel, Switzerland. Cells were grown in DMEM with 10% fetal calf serum (FCS) and 2 mM L-glutamine. The astrocytes were grown in ABM™ Basal Medium and AGM™ Bullet Kit® (Lonza International). All cells were grown in the presence of 100 units/mL penicillin, 100 µg/mL streptomycin, 12.5 units/mL nystatin (Biological Industries, Beit Haemek, Israel), and incubated in a humidified atmosphere of 5% CO_2_ and 95% air at 37°C.

Cell viability was measured with a FluoStar fluorometer using Hoechst fluorescent reagent at 390–460 nm [12].

### Syngeneic murine 4T1 breast carcinoma metastatic model

All animal experiments were conducted according to the NCI Laboratory Animal Care Guidelines with the approval of the Tel Aviv University Committee for Animal Experimentation and the Israel Ministry of Health (permit number M-05-024).

Eight- to ten-week old female BALB/c mice (Harlan, Jerusalem, Israel) were implanted subcutaneously (sc) with 4T1 mammary carcinoma cells (5×10^5^). Tumor length (L) and width (W) were measured twice weekly with a caliper, and volume was calculated according to the formula: (LxW^2^)/2.

Treatment commenced when the tumor volume reached 75–180 mm^3^ (∼10 days). The mice were randomly divided into 4 equal (n = 14/group) groups for treatment: vehicle control (saline); ip Dox 5 mg/kg once weekly; po AN-7 50 mg/kg 3 times weekly; ip Dox 5 mg/kg ip once weekly and po AN-7 50 mg/kg 3 times weekly (AN-7+Dox). Untreated, non-bearing tumor naïve mice were maintained under the same conditions. The experiment was terminated after 25 days. The tumors, lungs and hearts were harvested and weighed. Organs from 3 mice of each group were preserved for immunohistochemistry (IHC) and the remaining organs were frozen (−80°C) for Western blot analyses.

### Measurement of the Activity of HDAC classes I and II

Cells were seeded in 96-well plates (10×10^3^ cells/well, in quadruplicates) in growth medium for 24 h and then treated as indicated for 2 h in the presence of the HDAC substrate (Fluor de Lys™), the reaction was terminated by the addition of the Fluor de Lys™ developer (Biomol) and 2 µM trichostatin A (TSA). All of the above reagents were included in a kit for measuring activities of HDAC classes I and II (AK-503, Biomol, Plymouth Meeting, PA, USA). The % inhibition was calculated from the ratio of the fluorescence (measured at 355 nm excitation and 460 nm emission) in the drug treated compared to the untreated control culture.

### Western blot

Tumors and hearts were homogenized (Polytron; Kinematica, Lucerne, Switzerland) in lysis buffer [10]. Cells (2×10^6^ cells per plate) were plated in100 mm plates for 24 h then treated as specified and lysed. Protein levels in the samples were determined with the BCA protein assay kit (Pierce, Rockford, IL USA) and subjected to Western blot analyses with enhanced chemiluminescence to visualize γH2AX, HO-1, c-Myc, and TSP-1 bands [10]. The levels of HDAC1, HDAC2, HDAC5, Rad51 and MRE11 were determined using their specified primary antibodies followed by the secondary antibody IgG IRDye 680DX (LI-COR Biosciences). Each detected band was quantified using the Odyssey Infrared Imaging System (LI-COR Biosciences) and normalized to the level of actin in the corresponding lanes. The fold increase of a specific protein was determined by the ratio of the band intensity obtained from treated and untreated samples.

### Immunohistochemistry

Tumors and hearts were removed, fixed in 4% paraformaldehyde for 24 h, washed with PBS, dehydrated in increasing alcohol concentrations and embedded in paraffin. Paraffin-embedded blocks were processed as described [10]. Avidin-biotin nonspecific binding was prevented by a blocking kit, according to the manufacturer's protocol (Vector Laboratories, Burlingame, CA, USA). The sections were further incubated at 4°C overnight with the specified primary antibodies. The secondary antibodies were biotin conjugated goat anti-rabbit IgG (Santa Cruz). Slides were then stained with the ABC peroxidase system, developed with diaminobenzidine (DAB) or Nova Red chromogene substrates (Vector Laboratories) and counterstained with hematoxylin (Bio-Optica, Milano, Italy). The slides were examined using an Olympus BX 52 light microscope, and images were taken with an Olympus DP50 digital camera system.

### Picrosirius red staining

Slides of tumor and heart tissues were stained with 0.1% Picrosirius red and 0.2% fast green FCF (Sigma), as described [18].

### Data analysis

The average drug concentrations causing a 50% reduction in cell viability or HDAC activity (IC_50_) were determined from the formula of the best-fitted curve of percent survival or activity versus drug concentrations (≥3 independent dose-response titrations). A two-sided t-test between groups was performed using the Excel package for Windows 2003 (Microsoft). Time to treatment failure was estimated by Kaplan-Meir curves, and differences in time to treatment failure between groups were assessed with the log-rank test.

## Results

### AN-7 enhanced the anticancer efficacy of Dox and reduced its cardiotoxicity in 4T1 mammary carcinoma model

**Figure 1:**
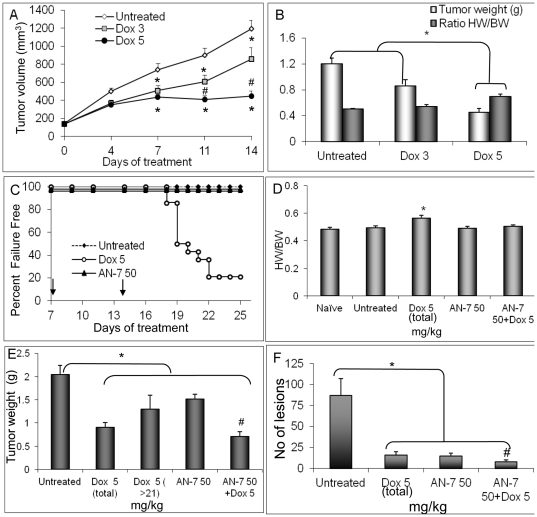
Effect of Dox, AN-7, and AN-7+Dox in the 4T1 murine mammary carcinoma model. (A) In the preliminary experiment, female Balb/c mice carrying 75–180 mm^3^ subcutaneous 4T1 tumors were randomly divided into three groups (10 mice/group) and were injected ip once a week with either vehicle, 3 or 5 mg/kg Dox. Tumor volume was measured twice a week up to day 14 when the experiment was terminated (after 2 Dox treatments). Mean±SE of tumor volume was: *p<0.05, untreated vs. the other groups; #p<0.05, Dox 5 mg/kg vs. Dox 3 mg/kg. (B) Mean±SE of tumor weight and the ratio of heart-weight to body-weight (HW/BW, g/g), measured at the experiment termination point were: *p<0.01, Dox 5 mg/kg vs. all other groups. (C) In the pivotal experiment, the Percent Failure Free mice bearing 75–180 mm^3^ subcutaneous 4T1 tumors, was assessed by a Kaplan-Mier graph. The mice were assigned to the following treatments: saline vehicle ip (n = 7) or po (n = 7); ip Dox, 5 mg/kg once/week (n = 14); po AN-7, 50 mg/kg 3 times/week (n = 14); po AN-7+ip Dox (n = 14). The arrows on the x-axis indicate the point in time of the second and the third Dox treatment. (D) Mean±SE of heart-weight to body-weight ratios (HW/BW, g/g) at the above experiment end-point was ^#^p<0.01, Dox 5 mg/kg vs. all other groups. (E) Tumor weight at the termination point (mean±SE) was: Dox 5 (total) represents the tumor weight of all the mice treated with 5 mg/kg Dox (n = 14), as measured at their individual end-points; Dox 5 (>21) represents the tumor weight of the mice (n = 5) treated with 5 mg/kg Dox and survived beyond day 21 of treatment. *p<0.02 all groups vs. untreated control; ^#^p<0.05 AN-7+Dox vs. AN-7 or Dox 5 (total) or p<0.01 Dox 5 (>21). (F) Mean±SE of lung lesions for the various treatment groups is shown. *p<0.02, untreated vs. all treatment groups; ^#^p<0.05, AN-7+Dox vs. AN-7 or vs. Dox.

In preliminary studies to determine the efficacy and toxicity range of Dox, mice were treated with saline or ip Dox at a dose of 3 or 5 mg/kg once weekly. We found that the 5 mg/kg dose of Dox inhibited tumor progression significantly better than the 3 mg/kg dose (p<0.05). On day 14, tumors and hearts were weighed and no difference in the mean body weight was detected among the three groups, however, a significant increase in heart-to-body-weight ratio (HW/BW) was measured in the 5 mg/kg Dox group (Figure 1A, B). On the basis of these results, we selected the dose of 5 mg/kg Dox to assess the anticancer and cardioprotective effects of AN-7 in the combined treatment (AN-7+Dox).

In the pivotal study, the treatment of the different groups was as specified in Figure 1, C–F. Treatment failure was defined by two criteria, a loss of ≥20% body weight or a tumor volume of ∼2 cm^3^. The experiment was terminated on day 25 of drug treatment when the mice of the control group failed. According to the first criterion, in the Dox-treated group, 50% (n = 7) of the mice failed the treatment on day 19, and 79% (n = 10) of them failed treatment on day 25. By contrast, in the other treatment groups, 100% (n = 14) of the mice survived on day 25, with acceptable body and tumor weights (Figure 1C).

The average HW/BW was significantly higher in the Dox-treated group than in the other groups, suggesting myocardial cardiomyopathy (p≤0.007; Figure 1D). When the same regimen of Dox was administered together with AN-7, there was no increase in the HW/BW and no morbidity. These observations support the notion that AN-7 imparted protection against Dox-induced cardiotoxicity thus increasing the animals' well-being.

Mean tumor weights at the experimental end-point were as follows: vehicle-treated (untreated), 2.05±0.2 g; Dox-treated (in the 4 surviving mice), 1.3±0.3 g; AN-7-treated, 1.52±0.1 g; AN-7+Dox-treated, 0.71±0.1 g. Mean tumor weight was significantly higher in the vehicle-treated group than in the other groups (p≤0.05; Figure 1E,) and significantly lower in the AN-7+Dox-treated group than in the other groups (p≤0.05; Figure 1E).

The lungs, removed from the euthanized mice, were stained in Bouin's solution and metastatic lesions were scored double-blindly. In the drug-treated groups the average numbers of lung lesions scored were: 15±4 in Dox-treated (n = 4 that survived to the experimental end-point); 14.9±3 in AN-7-treated (n = 14); 8.1±2 AN-7+Dox-treated (n = 14) and 87±20 (n = 14) in the vehicle treated mice. In the latter group the average number of lung lesions was significantly higher than in all of the treated groups (p≤0.001) and in the mice treated with AN-7+Dox it was the lowest (p<0.05, Figure 1F).

### The treatments' effects on HDACs expression and cellular activity in vitro

**Figure 2:**
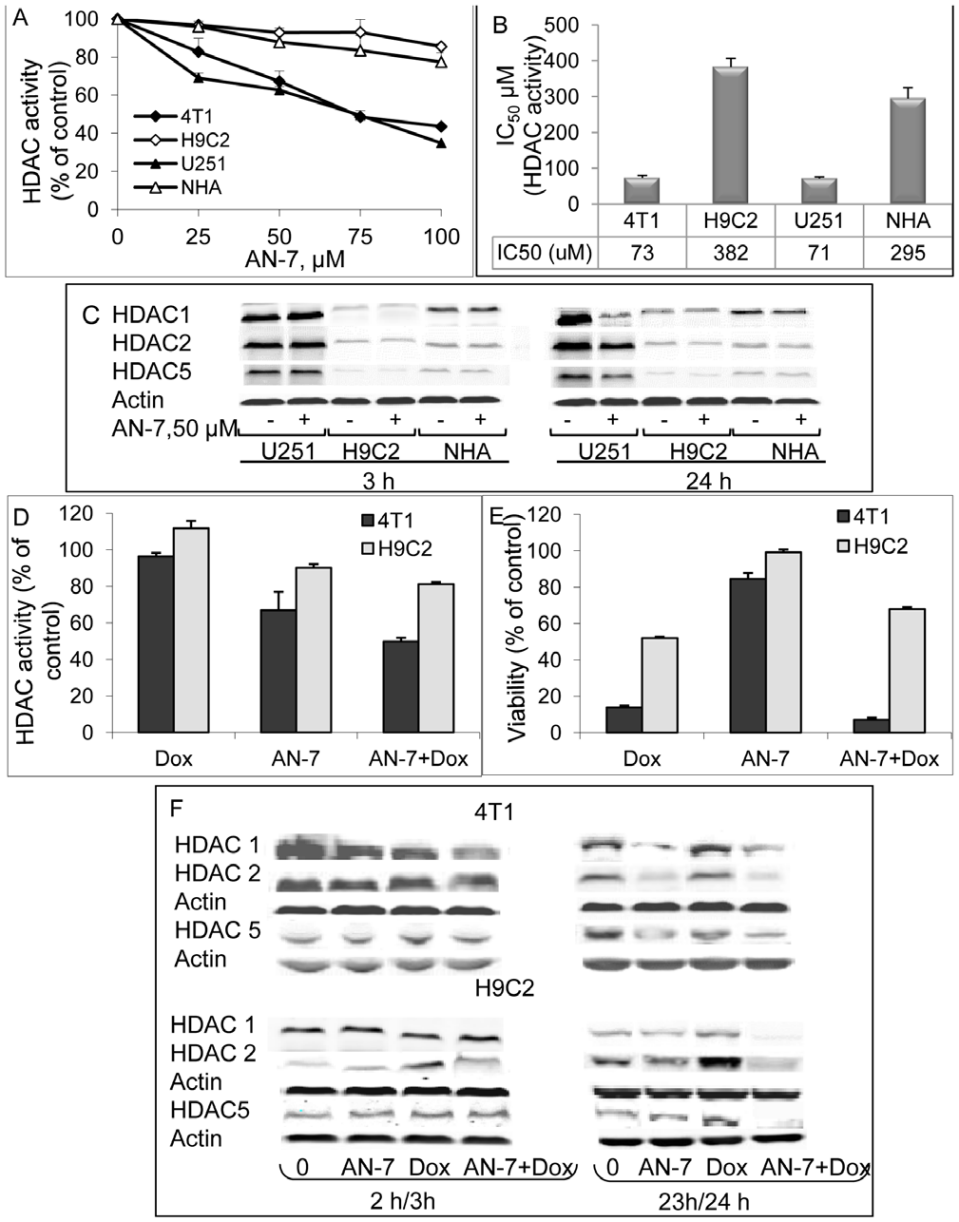
Effect of AN-7 on the activity and expression of cellular HDACs classes I and II. (A) The cells were exposed to AN-7 for 3 h in the presence of the HDAC fluorogenic substrate. The average % of HDAC activity as a function of 0–100 µM concentration of AN-7 of a representative experiment conducted in quadruplicates is shown. (B) The average IC_50_±SE of AN-7 for each of the cell types is calculated from three independent experiments. (C) Lysates of cells were loaded (35 µg protein/well) on 10% SDS gels and were subjected to Western blot analysis. HDAC1, HDAC2, HDAC5 or actin were detected with the appropriate antibodies. (D) HDAC activity of three experiments (in triplicate) conducted with 4T1 (cancer) and H9C2 (non-cancer) cells treated with AN-7 (50 µM) as a single agent for a total of 3 h, 1 h prior to the addition of the fluorogenic substrate and 2 h in its presence; Dox (200 nM) as a single agent treatment, or with the combination AN-7+Dox where 50 µM AN-7 was added 1 h prior to the addition of Dox and the fluorogenic substrate and then incubated for an additional 2 h. (E) The viability of the cells treated as described in (D) was determined by a Hoechst assay after 23 or 24 h for cells treated with Dox. (F) Cells were treated with AN-7 (3 or 24 h), Dox (2 or 23 h) or AN-7 (3 or 24 h)+Dox (2 or 23 h), as described (D). The lysates of the cells were subjected to Western blot analysis as described (C).

The direct effect of AN-7 on cellular HDAC activity was evaluated by incubating the cells for 2 h with the cell-permeable fluorometric substrate of HDAC classes I and II (Fluor de Lys™). The highest level of inherent HDACs activity (in untreated cells), measured in the human glioma U251 cells, was 100±2%. Relative to U251 cells, the activity in murine mammary carcinoma 4T1 cells was 86±1%, in the immortalized embryonic rat heart H9C2 cells, 37±2% and in NHA it was 23±2%. A dose-response of AN-7 HDACs inhibitory effect yielded in U251 cells an IC_50_ of 71±5 µM and in 4T1 cells an IC_50_ of 73±4 µM (Figure 2A). In H9C2 and NHA, 50% inhibition was unattainable and 100 µM of AN-7 inhibited less than 20% of the basal HDACs activity in both cell types. The estimated IC_50_ for H9C2 was 382±20 µM and for NHA was 295±30 µM (Figure 2 B). Overall, these data demonstrate that in the untreated cancer cells the basal activity of HDAC classes I and II is significantly higher than in the immortalized cells (H9C2) or in NHA (p< 0.0001). Moreover, the HDAC inhibitory potency of AN-7, as evident in the IC50 values, is at least 4-fold greater in cancer cells than in non-cancerous cells.

Treatment of U251, H9C2 cells and NHA with 50 µM AN-7 for 3 h showed (in a representative-reproducible experiment) that the highest expression levels of HDAC 1and 2 of class I and HDAC 5 of class II, were in U251 cells (Figure 2 C). These observations suggest that the activity of the HDACs in different cell types correlates with their level of expression. After 3 h of treatment, AN-7 had no noticeable effect on the expression of these enzymes therefore its inhibitory effect on their activity is likely to be a direct one. After 24 h, AN-7 downregulated the expression of these enzymes only in U251 but not in H9C2 cells or in NHA (Figure 2 C). Prolonged exposure of U251cells to AN-7, in addition to inhibiting these enzymes activity, also downregulated their expression.

Previously, we have shown that AN-7 synergistically increased Dox cytotoxicity against 4T1 cells, and conversely, it protected cardiomyocytes against Dox toxicity [12]. In an attempt to understand the basis for this disparity, we examined the manner by which AN-7, Dox and AN-7+Dox affect the total cellular activity of HDACs classes I and II in these cancerous and non-cancerous cells. Inhibition of total cellular HDACs activity in 4T1 cells was 33±5%, after 3 h with 50 µM AN-7, no inhibition after 2 h with 200 nM Dox and 50±2% after 3 h with AN-7+Dox. In H9C2 cells, the same treatment with AN-7 inhibited only 10±0.2%, Dox stimulated 12±0.4% and AN-7+Dox inhibited 19±0.2% of total cellular HDACs activity (Figure 2 D). The effect of the treatments on cell viability (data not shown) and HDAC 1, 2 and 5 expression (Figure 2 F) was barely discernible after the 2 or 3 h incubation. This is because 2–3 hours is too short a time period to detect the drugs' effects on viability or protein expression. We therefore, examined the survival and the expression of these enzymes after longer periods of treatment. Following 23 or 24 h treatments, the viability of 4T1 cells was as follows: AN-7, 85±3.2%, Dox 14±0.8% and AN-7+Dox 7±1.0% (Figure 2 E). A decrease in the expression of HDACs 1, 2 and 5 was observed in 4T1 cells treated with AN-7 or AN-7+Dox. Since Dox alone did not affect the expression of these enzymes, the reduction in HDACs expression in the combination treatment is due to AN-7 (Figure 2 F). A correlation between AN-7 induced inhibition of HDAC activity, reduction in HDACs expression and viability was observed. No correlation between the effect Dox on HDACs' activity, expression and viability was found. While Dox had a detrimental effect on the viability of these cells, it had no effect the cells' HDACs activity and expression.

Following 23 or 24 h treatments, the viability of H9C2 cells was: AN-7, 99±1.6%, Dox 52±0.8% and AN-7+Dox 68±1.2% (Figure 2E). In these cells AN-7 had no effect on cell viability and only a minor effect on HDAC activity and expression. Dox reduced viability and it noticeably increased the HDACs activity and expression (Figure 2 F). AN-7+Dox as compared to AN-7, reduced viability (p = 0.003), HDACs activity (p = 0.004) and expression. Compared to Dox treatment, the addition of AN-7, significantly increased viability (p = 0.004), reduced the elevated HDACs activity (p = 0.001) and expression.

### Effect of the treatments on HDAC1 and HDAC2 expression in vivo

**Figure 3:**
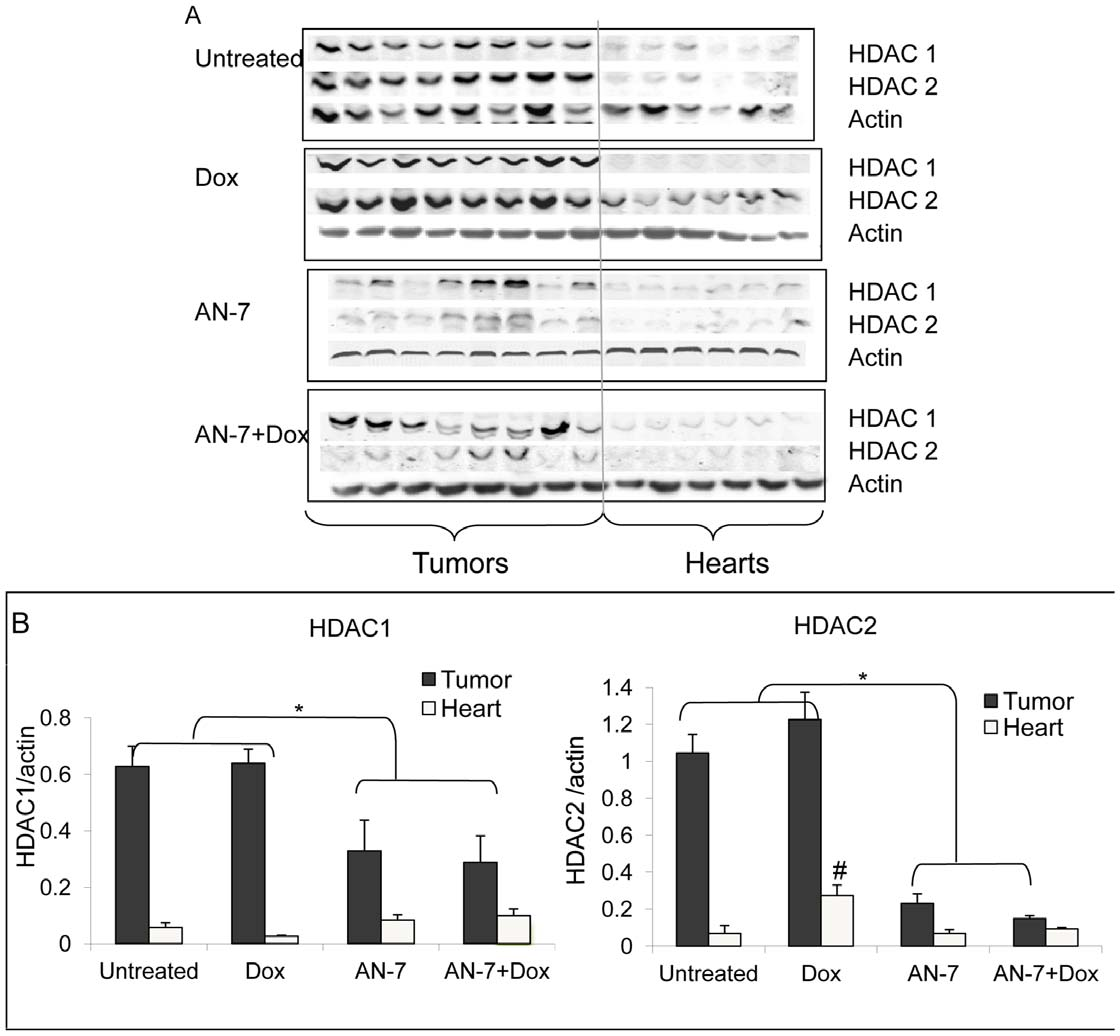
Effect of AN-7, DOX and AN-7+Dox on HDAC1 and HDAC2 expression in tumor or heart. (A) Extracts of tumors from 8 mice and hearts from 6 mice per treatment group were loaded (20 µg protein/well) on 10% SDS gels, and subjected to Western blot analysis. HDAC1, HDAC2 or actin were detected with the appropriate antibodies. (B) Expression of HDAC1, HDAC2 or actin in tumors and hearts was analyzed by Odyssey 2.1. The intensity ratio of HDACs to actin (mean±SE) is presented. *p<0.05, tumors of untreated or Dox-treated vs. AN-7 or AN-7+Dox; ^#^p<0.007, HDAC2 expression in the hearts of Dox treated vs. all other treatment groups.

The levels of HDAC-1 and HDAC-2 were 10–15-fold higher in the tumors than in the hearts (Figure 3). The levels of HDAC-1 and HDAC-2 were unchanged in the tumors of Dox-treated mice. Whereas, HDAC1 levels decreased 2-fold in the AN-7 and the AN-7+Dox-treated groups. HDAC2 levels decreased ∼5-fold in the AN-7 group and ∼7-fold in the AN-7+Dox-treated group. In contrast to the downregulation of the HDACs expression by AN-7 in the tumors, AN-7 and AN-7+Dox did not affect the expression levels of HDAC1 or HDAC2 in the heart tissue. Similarly to the observation in vitro, Dox upregulated HDAC-2 by ∼4-fold, an effect attenuated by the addition of AN-7 to the Dox treatment.

### Effects of the treatments on viability markers and DNA damage and repair in vivo

**Figure 4:**
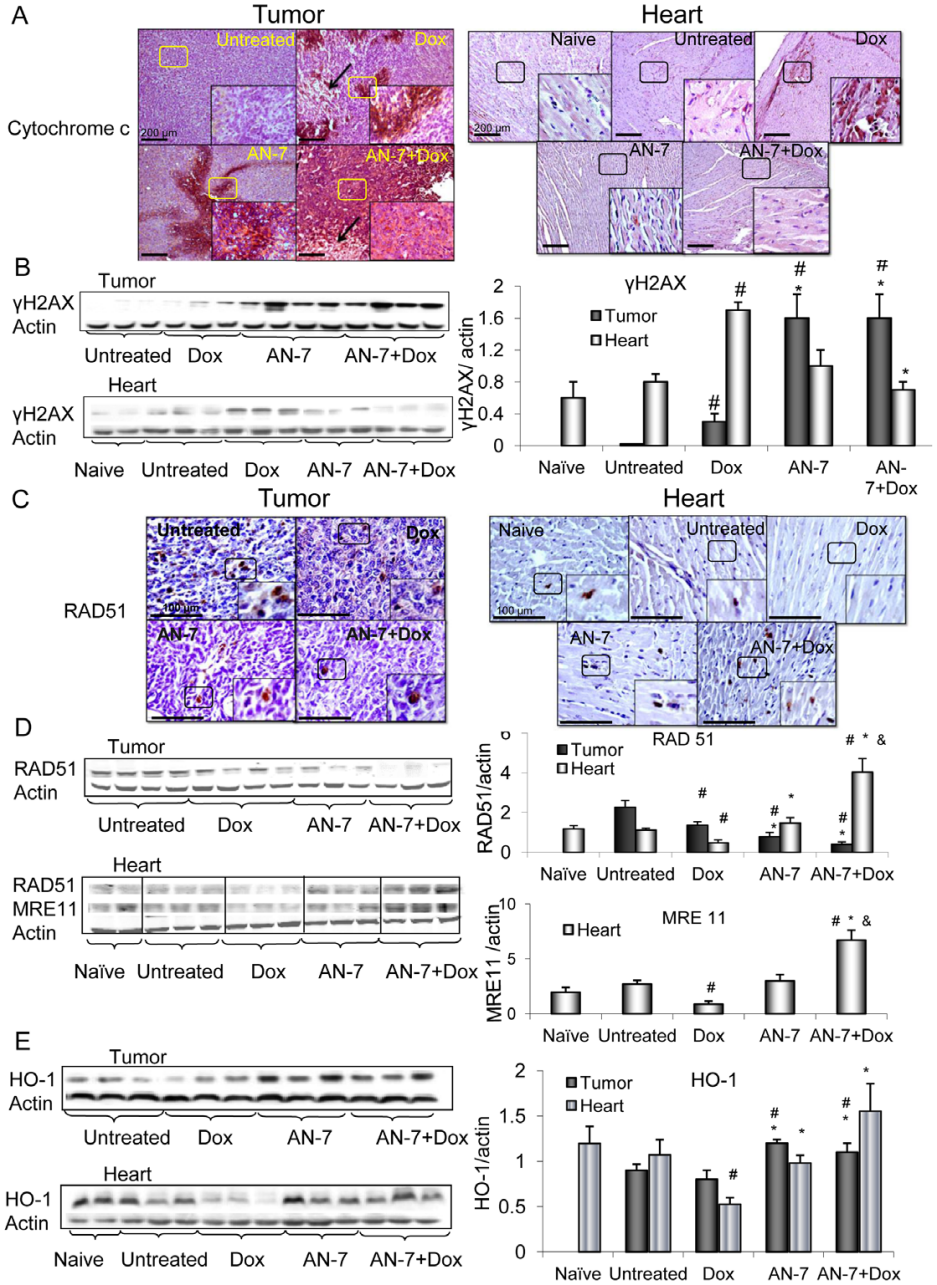
Effect of AN-7, DOX and their combinations on apoptosis, DNA damage and repair and HO-1. Sections of tumors (left hand-side) and hearts (right hand-side) from mice were stained with the specified antibodies and counter stained with hematoxylin. For the detection of markers by Western blot analysis, the samples were resolved on SDS gels. Lysates of tumors (20 µg protein) or hearts (40 µg protein) were loaded on the gels (left hand-side). Mean±SE of the ratios of bands intensity, each normalized to actin, is shown for tumors (n = 7), hearts (n = 7) and for 4 hearts of naive mice (the right hand-side). (A) IHC with anti cytochrome c (Nova Red). The arrows mark the necrotic areas; Bar = 200 µm, the 4-fold magnified picture in the insert was taken from the area indicated by the square. (B) 15% SDS gel stained for γH2AX; (C) IHC with anti Rad51 (DAB); Bar = 100 µm, the 2-fold magnified picture in the insert was taken from the area indicated by the square. (D) 10% SDS gel stained for Rad51 and MRE11; (E) 12% SDS gel stained for HO-1. ^#^p<0.05 vs. untreated control; *p<0.05, Dox vs. AN-7 and AN-7+Dox.

The expression of proteins associated with cell survival and death were monitored by IHC and or by Western blot analyses. Cytoplasmic cytochrome-c, a marker of apoptosis, was visible as positively stained patches in the tumors of mice treated with AN-7 or Dox, in tumors of mice treated with AN-7+Dox the staining was intensified. In tumors of Dox- and AN-7+Dox-treated mice, in addition to apoptotic area, necrotic areas (marked with arrows) were also visible. No detectable levels of cytoplasmic cytochrome-c or necrosis were found in the tumors of the vehicle-treated mice. In the hearts, cytoplasmic cytochrome-c was detected only in the Dox-treated mice. Addition of AN-7 to Dox treatment abrogated it, demonstrating that AN-7 protects against Dox cardiotoxicity (Figure 4A).

To assess DNA damage, we evaluated changes in the levels of γH2AX, a marker of DSBs. In the tumors, all the treatments induced an increase of γH2AX, however, the greatest increase was observed after AN-7+Dox treatment (Figure 4B). In the hearts, Dox increased γH2AX levels significantly as compared to all other treatments (Figure 4B).

The expression of Rad51, a marker of homologous recombination-guided DNA repair of DSBs [19], was abundant in the tumors and scarce in the hearts of vehicle-treated mice. Western blot analysis and IHC showed that in the tumors, all the treatments suppressed Rad51 expression, while in the hearts, the low levels of Rad51 expression was suppressed only by Dox (Figure 4 C and D). The level of Rad51 expression in the tumors decreased by AN-7−, Dox- and AN-7+Dox treatments by approximately 3-, 2- and 6- fold, respectively. The effect of the combined treatment on Rad51 was significantly greater than that of each of the drugs alone (p≤0.03, Figure 4D). These observations demonstrate that the drugs, in addition to inducing DNA damage, further intensify this insult by suppressing the repair mechanism. In cardiac tissue, the addition of AN-7 to Dox resulted in an increase of Rad51 expression (∼8 fold, p = 0.0003).

Another protein whose function is maintaining genomic stability and DNA repair is MRE11 [20]. This protein was expressed in the hearts of the mice bearing 4T1 tumor, but not in their tumors. MRE11 basal expression level was similar in the hearts of naïve, vehicle-and AN-7 treated mice. Dox treatment downregulated MRE11 expression by 2- fold and the addition of AN-7 to Dox, significantly upregulated it to a level which was higher than its basal level of expression (p = 0.001, Figure 4D). Both RAD51 and MRE11 expression were significantly higher in the hearts of mice treated with Dox+AN-7 compared to all other treatments. This observation strongly supports the notion that under stress conditions, AN-7 imparts its protective activity by inducing DNA repair pathways.

### Effects on HO-1 expression

HO-1 was shown to suppress growth, invasion and migration in cancer cells, while in the myocardium it confers protection [12], [16]. HO-1 expression was similar in the tumors of vehicle- and Dox-treated mice, and increased significantly in tumors of the AN-7− and AN-7+Dox-treated mice (p = 0.001 and p = 0.03, respectively). In the heart, HO-1 levels dropped ∼2-fold in the Dox-treated mice compared to the naïve, vehicle-treated or AN-7-treated mice (p≤0.03). The addition of AN-7 to Dox increased HO-1 expression ∼3-fold compared to Dox treatment alone (p = 0.006). These observations suggest that AN-7 in the combination treatment induces a protective response against the oxidative stress imposed by Dox (Figure 4E).

### Effect of the treatments on Ki-67, c-Kit, and c-Myc expression

**Figure 5:**
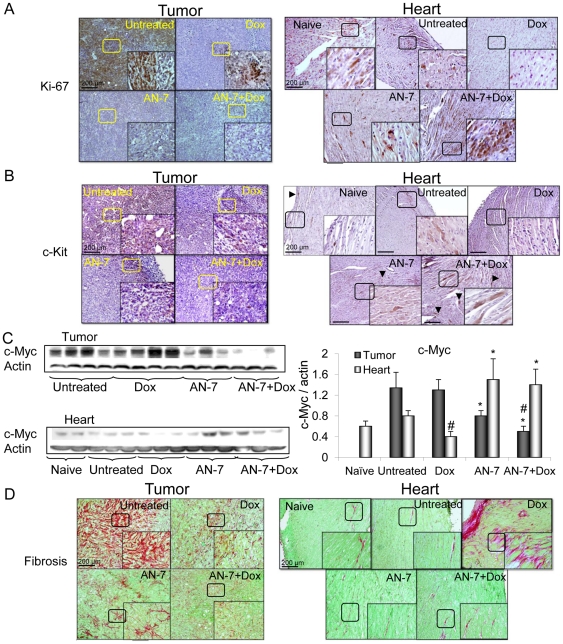
Effect of AN-7, DOX and their combinations on markers of proliferation and fibrosis. Sections of tumors and hearts were stained (DAB) for: (A) Ki-67; (B) c-Kit and both were counter stained with hematoxylin. The arrowhead marked the c-Kit positively staind cells. Bar = 200 µm, the 4-fold magnified picture in the insert was taken from the area indicated by the square. (C) Lysates samples, as described above, were resolved on 10% SDS gel and stained for c-Myc (left). The Mean±SE ratio of c-Myc/actin expression is shown (right). ^#^p<0.05, as specified, vs. untreated control; *p<0.05, as specified, vs Dox. (D) Picrosirius red and fast-green for the visualization of interstitial fibrosis. Bar = 200 µm, the 2-fold magnified picture in the insert was taken from the area indicated by the square.

The high levels of Ki-67 and c-Kit in tumors from untreated mice, signifying undifferentiated proliferating cell populations, were substantially reduced after treatment with Dox or AN-7 and were abrogated after treatment with AN-7+Dox (Figure 5A, B).

In hearts of mice treated with vehicle or AN-7, Ki-67 and c-Kit expression levels, were similar to the basal expression level found in naïve mice. Treatment of mice with Dox alone, downregulated their expression, whereas, the addition of AN-7 to Dox treatment, elevated Ki-67 and c-Kit expression to the level which was noticeably higher than their basal expression level (Figure 5A, B).In the naïve, vehicle- and AN-7− treated mice c-Kit positive cells were located mainly in the outer regions of the ventricular wall, whereas, in AN-7+Dox treated hearts, foci of c-Kit positive cells were dispersed throughout the ventricular myocardium.

The protein c-Myc is an important regulator of normal cell physiology and of tumor cell oncogenic pathways. The normally high tumor expression of c-Myc was unaffected by Dox but decreased after AN-7 treatment and further decreased following AN-7+Dox treatment. The expression of c-Myc was lower in the hearts than in the tumors, and Dox treatment decreased it further, whereas AN-7 alone or in combination with Dox increased it markedly (Figure 5C).

### Effect of the treatments on fibrosis

In the tumors stained with Picrosirius red to label fibrous collagen, extensive fibrosis detected in the vehicle-treated group was substantially reduced in the Dox- and AN-7-treated groups, and dramatically diminished following treatment with AN-7+Dox (Figure 5D). Since interstitial fibrosis plays a vital role in maintaining the supportive microenviroment of the tumor [21], its disruption by AN-7, Dox and AN-7+Dox imposes a restriction on tumor progression.

The hearts were largely negative for Picrosirius red in the naïve, vehicle-treated, and AN-7-treated groups. However, in the Dox-treated group, highly positive staining was visible in the myocardium indicating interstitial fibrosis, a hallmark of cardiomyopathy [22]. The response to Dox was attenuated with the addition of AN-7 to Dox treatment.

### Effect of AN-7 or its combination with Dox on angiogenesis

**Figure 6:**
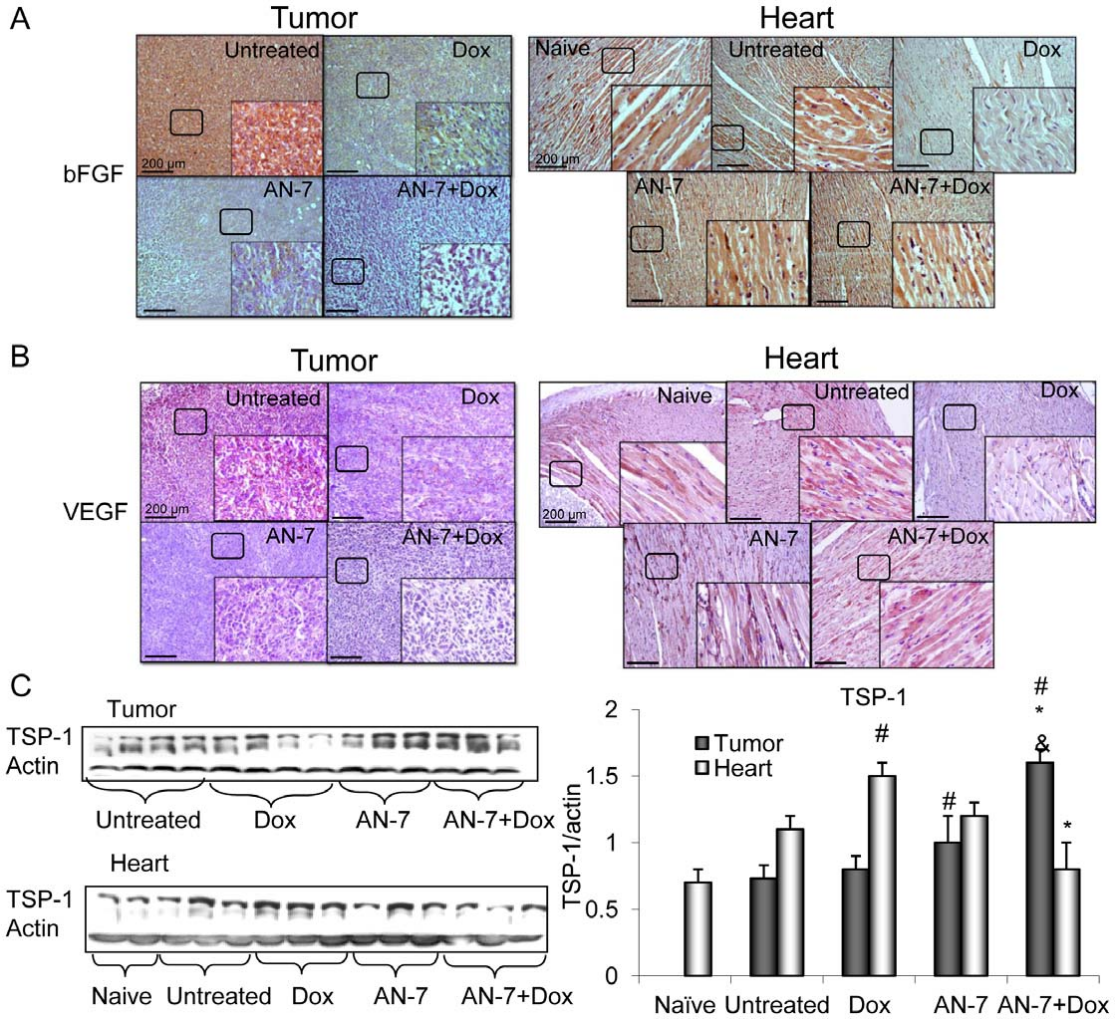
Effect of AN-7, DOX and their combinations on angiogenesis. Sections of tumors and hearts were stained for (A) lo-FGF (DAB); (B) VEGF (Nova Red) and counter stained with hematoxylin. Bar = 200 µm, the 4-fold magnified picture in the insert was taken from the area indicated by the square. (C) Lysates samples (as described above) were resolved on 7.5% SDS gels and stained for TSP-1(left). Mean±SE of the ratio of TSP-1/actin expression is shown (right), ^#^p<0.05, as specified, vs. untreated control; * p<0.05, as specified, vs. Dox; ^&^p<0.05, AN-7+Dox vs. AN-7.

Low molecular weight fibroblast growth factor-2 (18 kDa, lo-FGF-2) and vascular endothelial growth factor (VEGF) are potent angiogenic stimulators [16]. Levels of lo-FGF2, and VEGF were high in the tumors from the vehicle treated mice (Figure 6A, B). They decreased after treatment with Dox or AN-7 and were abrogated after treatment with AN-7+Dox. This suppression indicates that in the tumors, AN-7 and Dox exerted antiangiogenic activities that were further intensified by the combination of the two agents. The upregulation of TSP-1 leads to an inhibitory effect on tumor angiogenesis that may slow tumor growth [23]. As shown in Figure 6C, the expression level of TSP-1 was similar in tumors from the vehicle and Dox-treated mice, increased significantly in AN-7-treated mice (p = 0.03), but increased still further in the AN-7+Dox treated mice (AN-7 vs. AN-7+Dox, p = 0.01). This suggests that the reduction in angiogenesis in the tumor is the outcome of a reduction in pro-angiogenic and an increase in anti-angiogenic factors.

In naïve and vehicle-treated mice hearts, the expression of the two angiogenic markers, lo-FGF-2 and VEGF, was indistinguishable. It increased with AN-7 treatment, decreased with Dox treatment, and was restored to normal levels by treating with AN-7+Dox (Figure 6 A, B).

In line with the angiogenic effect of AN-7 in the heart, the expression levels of TSP-1 in naïve, vehicle or AN-7 treated mice, was similar, whereas in hearts of Dox treated mice there was a significant increase. The addition of AN-7 to this treatment significantly reduced the TSP-1 levels. The latter demonstrates that the addition of AN-7 prevents suppression of angiogenesis by Dox.

## Discussion

Our group previously demonstrated the protective effect of AN-7 against Dox-toxicity in cardiomyocytes and astrocytes and AN-7 augmentation of Dox's anticancer activity against breast carcinoma and glioblastoma cells in vitro [12], [15]. The aim of the present study was to substantiate our earlier findings in vivo and to investigate the underlying cellular and molecular modifications responsible for this disparity. In this study, the tissue-specific response of AN-7 was supported by the different concomitant physical and molecular changes that took place in the tumors and hearts of Balb-c mice bearing sc 4T1 mammary tumors, following treatments with AN-7, Dox and their combination.

Dox treatment caused a dramatic decrease in mice body weight, leading to the experimental toxicity endpoint significantly earlier than in the mice treated with vehicle or AN-7. However, when AN-7 was added to the Dox treatment, it prolonged the time to failure of the mice, indicating AN-7's protective role against Dox-induced toxicity. At the same time, AN-7 increased the anticancer efficacy of Dox, as seen by the significantly stronger inhibition of tumor growth and reduction of lung metastases. Thus, the combined treatment was more effective than treatment with either drug alone. The protective effect of AN-7 against Dox-induced toxicity took place at the same time as its potentiation of the antineoplastic efficacy of Dox. These findings are consistent with our previous in vitro studies [12], [15]. Since Dox is used to treat breast cancer and other type of cancers, the AN-7+Dox combination is potentially beneficial for these protocols [13], [14]. Due to AN-7's dual mode of action in vivo, we consider it a valuable tool for the investigation of specific molecular events, which occur concomitantly in the milieu of the normal and in cancerous tissues.

We noted striking differences in the inherent cellular activity of HDACs classes I and II and also in the level of expression of HDAC1, HDAC2 and HDAC5 between non malignant and malignant cells. The inherent activities of these HDACs in astrocytes and H9C2 cardiomyoblasts were significantly lower compared to those found in cancerous cells. AN-7 exhibited a cell-type-specific inhibition of classes I and II HDAC activity. It inhibited HDAC activity in U251 and 4T1 cells after 3 h of treatment to a significantly greater degree than it inhibited this activity in the immortalized H9C2 or astrocytes. The levels of HDAC 1, 2, 5 expression in U251 cancer cells did not change after 3 h of treatment with AN-7, but decreased after 24 h. In astrocytes and H9C2 cells, AN-7 did not change the level of expression of these HDACs after 3 or after 24 h of treatment. Taken together, these data indicate that AN-7 affects the activity of classes I and II HDACs as well as the expression of representative members of HDAC class I (HDAC 1, 2) and HDAC class II (HDAC 5), specifically in cancer cells. In light of these results, the anticancer efficacy and the selectivity of AN-7 may be ascribed to its specific suppression of HDACs activity and expression, as well as to the disparity in the inherent HDAC activity in the different cell types. These results substantiate the notion that AN-7 specifically targets the elevated HDACs activities and expression in cancer cells.

Correspondingly, the 10–15-fold lower levels of HDAC1 and HDAC2 in the hearts compared to the 4T1 tumors were unaffected by Dox but were suppressed by AN-7 and AN-7+Dox. These observations are in accordance with reports that high levels of class I HDACs are expressed in tumors [24]. Since the high level of HDACs expression play a role in sustaining the oncogenic state and correlate with disease aggressiveness [25], [26], [27], we may infer that AN-7 exerts its anti-cancer activity, at least in part, by downregulating the activity and expression of HDACs in the tumors.

Although AN-7 treatment alone did not affect HDAC expression in the myocardium, when it was added to Dox, it negated the Dox-induced increase in HDAC-2 expression. This apparent discrepancy (seen also in vitro) may be explained by reports that the activation of HDAC-2, but not HDAC-1, plays a critical role in the development of cardiac toxicity [3], [28]. Thus, AN-7 downregulation of HDAC-2 expression may be part of the mechanism whereby it protects cardiac cells against Dox [29].

Altogether, these findings support the notion that the discriminatory effect of AN-7 stems also from its ability to affect the HDAC expression and activity in a cell-type specific manner. The caveat is that this conclusion is based on the examination of only classes I and II out of the four known classes of HDACs [27]. The relevance of distinct HDAC repertoires to the specificity of AN-7 action in normal and cancerous tissues remains to be further studied.

The interplay among the pro- and anti- survival and angiogenic factors in the tumor and the myocardium showed tissue selective differences. Dox induces formation of DNA DSBs by elevating cellular ROS levels, intercalation into the DNA, the formation of Dox-DNA adducts, and by poisoning the transient topoisomerase II-DNA intermediate formed during transcription and replication [30], [31], [32]. In cancer cells, the AN-7 induction of ROS probably leads to DSBs formation and when added to Dox, the effect is further enhanced [12].

In response to DSBs formation, γH2AX accumulates on the site and interacts with a multi-protein complex that activates a repair-control response involving Rad51 and MRE11 [20], [33]. An effective repair pathway is associated with cell survival, and conversely, a repressed pathway leads to cell death. We found that treatment of mice with Dox, AN-7, and to a greater degree, AN-7+Dox simultaneously increases DNA damage and decreases DNA repair, leading to cancer cell mortality, as manifested by the increased levels of cytoplasmic cytochrome-c.

In the hearts, Dox treatment induced the expression of γH2AX and reduced the expression of Rad51 and MRE11, thereby suppressing DNA DSBs repair causing the release of cytochrome c to the cytoplasm thus leading to cell death. AN-7 alone had no myocardial cell damage, but its addition to Dox, concurrently decreased the DNA damage and increased the DNA repair, thereby, negating the release of cytoplasmic cytochrome-c. This sequence of events contributes to the protective effect of AN-7 against Dox cardiotoxicity, and is in agreement with our previous studies [12], [15].

AN-7+Dox treatment led to opposing changes in the DNA damage-response mechanism within the tumor and the heart of the same mice. In the tumor, the DNA damage was intensified and the DNA repair was suppressed, culminating in cell death. Conversely, in the heart, DNA damage was decreased and DNA repair was enhanced, preserving the viability of the cells in the heart. As mentioned above, we attribute the cell specificity of AN-7 to its inhibitory effect on distinct HDACs. This is consistent with recent reports demonstrating the involvement of histone selective acetylation in the activation of DNA repair [34], [35]. Taken together, the opposing effects of AN-7 in the tumor and the heart infer that DNA damage and response mechanism are selectively targeted by AN-7.

HO-1 has been shown to inhibit cancer cell proliferation and reduce tumor invasiveness [36]. In the present study, AN-7 and AN-7+Dox caused a significantly greater increase in HO-1 expression than Dox alone, suggesting that increased HO-1 expression is one of the molecular changes involved in the anti-proliferative and anti- metastatic activities of these treatments.

In the heart, HO-1 functions under stress conditions, when it rapidly degrades pro-oxidant heme to carbon monoxide (CO) and biliverdin/bilirubin. CO inhibits inflammation and oxidative-stress [37], and the biliverdin/bilirubin reduces oxidative activity of the released Fe^2+^ [38]. Together, these events result in protection of the heart [39]. We found that treatment with AN-7+Dox significantly increases HO-1 expression compared to Dox alone, suggesting that AN-7+Dox induces a defensive response against the oxidative stress and inflammation imposed by Dox. These observations are in line with our earlier findings that AN-7 reduced the elevation of ROS in cardiomyocytes, and of TNF-α and interferon-γ in the heart and plasma of mice treated with a high dose of Dox [12].

Fibrosis in tumors provides the supportive extracellular matrix that is secreted by activated myofibroblasts [21]. Growth factors secreted by cancer associated fibroblasts, stimulate tumor cell proliferation and increase angiogenesis and tumor cell invasion [40], [41]. Fibroblasts were shown to be key modulators of immune polarization in the tumor microenvironment of the 4T1 murine model and therefore, are proposed to be valid targets for metastatic breast cancer therapy [42]. In an earlier study, we demonstrated that AN-7 inhibited the migration of prostate cell lines and the metastasis and angiogenesis of prostate tumors to the lungs and the bone-marrow [8], [10]. In the present study, we demonstrate that treatment of tumor-bearing mice with AN-7+Dox, as compared to each drug alone, dramatically decreases the abundance of the fibroblasts in the tumor. This was accompanied by a reduced deposition of collagen and a suppression of angiogenesis. This was achieved by a reduction in pro-angiogenic factors and an increase of anti-angiogenic factor (TSP-1), thereby limiting the capacity of the tumor to grow.

Previously, we have shown that the viability of cardiofibroblasts was dramatically repressed by AN-7+Dox, suggesting that in the heart, AN-7 may attenuate Dox-induced fibrosis [12]. Heart fibrosis is a classical feature of tissue remodeling in a response to a variety of pathological conditions. Heart fibrosis is characterized by the expansion of the extracellular matrix and in particular the accumulation of collagen type I [22]. HDACIs were reported to reduce the deposition of fibrotic extracellular matrix by inhibiting myofibroblast proliferation [41], [43]. The above is supported by our present study that unequivocally demonstrates that the addition of AN-7 to Dox treatment negates Dox-induced fibrosis in the heart. This finding has important implications for the preservation of the heart elasticity and contractility. Taken together, cancer associated fibroblasts and the cardiofibroblasts are another target of AN-7 activity.

The protein Ki-67 is a marker for cancer and normal cell proliferation [44]. The tyrosine kinase receptor c-Kit activates downstream pathways leading to cell proliferation and survival [45]. Overexpression of c-Kit in tumors points to the presence of cancer stem cells and predicts treatment resistance and poor outcomes. Therefore, disruption of c-Kit expression in c-Kit-driven tumors represents a promising therapeutic approach [46], [47]. In this study we demonstrated that both Dox and AN-7 substantially reduce Ki-67 and c-Kit expression in the tumor and AN-7+Dox abolish it, supporting the notion that the combination treatment can potentially benefit treatment-resistance and aggressive tumors.

In the heart, Ki-67 and c-Kit were shown to be expressed by cardiac progenitor cells (CPC) residing mainly in the epicardium [48], [49]. In the present study, the prevalence of Ki-67 and c-Kit expressing cells in the naïve, vehicle and AN-7 treated hearts, was similar. The change in locality of the c-Kit positive cells is noteworthy. In naïve, vehicle and AN-7- treated mice, they resided mostly in the outer regions of the myocardium. Following Dox treatment their expression was diminished and with the addition of AN-7 to Dox, their expression was restored as foci distributed throughout the myocardium. These foci may be regeneration sites repopulated with CPCs. Since treatment with Dox was reported to induce CPC death, AN-7 protection of the c-Kit positive cells is likely to preserve the CPCs and therefore the cardio-regeneration capacity [48], [50], [51]. Angelis et al. suggested that Dox-induced depletion of the CPC pool within the myocardium contributes to the dramatic manifestation of heart failure in animal models and humans due to the diminished regenerative capacity of their hearts [51]. Patients, who survive cancer treatment with Dox, face the risk of developing heart failure, either soon after treatment or later in life. Therefore, the addition of AN-7 to Dox treatment is a promising approach to the reduction of cardiac complications in these patients.

The protein c-Myc is aberrantly overexpressed in more than 30% of all cancers, including 4T1 cells [52]. In the 4T1 tumors, its expression was unaffected by Dox, significantly downregulated by AN-7 and suppressed further by AN-7+Dox. The repression of c-Myc is associated with anti-angiogenic effects marked by an increased expression of the potent angiogenesis inhibitor TSP-1, repression of the pro-angiogenesis factors, lo-FGF-2, and VEGF. TSP-1 acts as a scavenger of matrix-associated angiogenic factors and is repressed by c-Myc, which upregulates its repressor miR17-92 [53], [54]. On the basis of these findings, we propose that the suppression of c-Myc plays a central role in the inhibition of tumor angiogenesis.

Coupled to these changes, the myocardial c-Myc expression increased significantly by AN-7 alone and decreased with Dox treatment. The addition of AN-7 to Dox treatment reversed the decrease in c-Myc expression. The basal level of TSP-1, the c-Myc-regulated angiogenesis inhibitor, was unaffected by AN-7 and increased by Dox. The addition of AN-7 to Dox treatment relieved the myocardium from TSP-1 angiogenic repression, as was seen by co-elevation in the expression of lo-FGF-2 and VEGF. These observations indicate that AN-7 also elicits its cardioprotective activity by increasing pro-angiogenic factors in the heart.

Collectively, our findings support the notion that AN-7 affects cancer and normal cells differently. Moreover, we have shown that in addition to disparately affecting HDACs activity and expression, AN-7 and AN-7 in combination with Dox, distinctively modulated major molecular and cellular events affecting cell survival.
